# Person-centred care as an evolving field of research: a scoping review

**DOI:** 10.3389/frhs.2025.1534178

**Published:** 2025-04-04

**Authors:** Emma Forsgren, Caroline Feldthusen, Sara Wallström, Lovisa Thunström, Lars Kullman, Richard Sawatzky, Joakim Öhlén

**Affiliations:** ^1^ Institute of Health and Care Sciences, Sahlgrenska Academy, University of Gothenburg, Gothenburg, Sweden; ^2^ University of Gothenburg Centre for Person-Centred Care (GPCC), Sahlgrenska Academy, University of Gothenburg, Gothenburg, Sweden; ^3^ Department of Occupational Therapy and Physiotherapy, Sahlgrenska University Hospital, Gothenburg, Sweden; ^4^ Department of Forensic Psychiatry, Region Västra Götaland, Sahlgrenska University Hospital, Gothenburg, Sweden; ^5^ Centre for Ethics, Law and Mental Health (CELAM), University of Gothenburg, Gothenburg, Sweden; ^6^ Gothenburg University Library, University of Gothenburg, Gothenburg, Sweden; ^7^ School of Nursing, Trinity Western University, Langley, BC, Canada; ^8^ Centre for Advancing Health Outcomes, Providence Health Care, Vancouver, BC, Canada; ^9^ Palliative Centre, Sahlgrenska University Hospital, Gothenburg, Sweden

**Keywords:** systematic review, scoping review, patient-centered care, person-centred care, text mining, EPPI-Reviewer, literature review as topic, integrated care

## Abstract

**Introduction:**

Changes in policy towards a healthcare approach viewing patients as persons provide calls for person-centred healthcare practices. The objective of this scoping review was to present an overview of the international literature on PCC.

**Methods:**

Database-specific search string including index terms and free text words related to PCC were constructed to identify relevant literature indexed in PubMed, Scopus, PsychINFO, CINAHL and Web of Science. Two different methods of combined manual and computer-assisted screening were applied to identify citations to be included in the review.

**Results:**

In total, 1,351 publications were included, whereof theoretical and empirical studies were most prevalent in the sample. For the latter, the most common setting was hospital care. The study population was most often health professionals or patients. The most frequently used term was patient-centred, followed by person-centred and family-centred. Research from six continents was included. An exploration of collaborations and research clusters has revealed several clusters.

**Discussion:**

This review provides a snapshot of the literature on PCC. The lack of clarity in terminology presents barriers to comprehensively overviewing the vast amount of available research within the field, which in turn presents challenges for research-based policy and practice development.

## 1 Introduction

In recent decades there has been increasing demand for patients' perspectives to be taken into consideration when organising and carrying out healthcare. These demands have come from different stakeholders, including patient and family member organisations, healthcare professionals, researchers, and policymakers (1). A healthcare approach viewing the patient as a person, emphasizing co-creation and partnerships between patients and professionals, has become the gold standard of care within the healthcare sector. This approach, which we will henceforth refer to as person-centred care (PCC), can be understood from the perspective of different frameworks (2–6). The implementation of PCC has been proposed as a way of improving quality of care and is included in European regional policy (7), as well as global healthcare policy (8). Research shows promising effects, such as increased effectiveness, patient satisfaction and cost reduction (9).

Due to the variety of people involved with similar but not identical starting points and goals, a plethora of different terms denoting this field of study have arisen—terms such as the aforementioned person-centred care, as well as patient-centred care, people-centred care, family-centred care etc. (10). Sometimes, different terms are used to label the same construct, while at other times, the same term is used to refer to different constructs. While some researchers assert that the conceptual differences between constructs are minor, others view the end goal of care as different, for example, when looking at patient and person-centred care (11, 12). The link between the term used and the basis of, for example, a PCC intervention is often not clear in research today (10).

Apart from the above inconsistencies in conceptualizations and terminology, the boundaries of this research field are blurry and there is an evident overlap with other fields, such as research solely focused on shared decision-making or research on patient and public involvement (PPI). In addition, only one medical subject heading (MeSH) currently exists, i.e., patient-centred care, further adding to problems with delimitation. This heading was introduced to PubMed in 1995 and is available in the MeSH tree syntax under primary care and narrative medicine (13). Patient-centred care is defined as: “Design of patient care wherein institutional resources and personnel are organized around patients rather than around specialized departments”, hence not encompassing the conceptualization or delivery of care.

To stay within project constraints, the mentioned challenges may result in reviews choosing a limited scope, using only one or a few terms, having a short time frame or focusing on a specific population or healthcare context. While pragmatic, this approach risks providing an incomplete overview of the research field. For instance, two current reviews, a white paper and an edited volume, all on PCC (9, 14–16), have minimal overlap in the included studies, suggesting that different domains of PCC research are being presented. This example of different domains in the targeted field also raises the question of whether there are active collaborations between researcher groups or whether we are working in separate silos. If so, this could be an obstacle to building a shared knowledge base from which to generate research and evidence-based policy in the long run.

Thus, the objective of this scoping review is to present an overview of international literature on PCC and to answer the following research questions: (1) What populations, settings, research approaches, and designs are represented in PCC literature, (2) Which terms and keywords are used in PCC literature, and (3) Can research collaborations and clusters be observed in the research field of PCC?

## 2 Methods

### 2.1 Study design

A scoping review methodology combined with bibliometric analysis was identified as the best approach for describing the vast amount of literature on the topic of PCC, which has not been thoroughly examined or, is characterized by complexity and heterogeneity (17). The methodology outlined by Arksey and O'Malley (18) and Levac (19) involves five key phases: (1) identifying the research question, (2) identifying relevant studies, (3) study selection, (4) charting the data, and (5) collating, summarizing, and reporting. We followed the Preferred Reporting Items for Systematic Reviews and Meta-Analysis statement (PRISMA-P) (20) and PRISMA-Scr extension for scoping reviews (21). The review has been registered in PROSPERO ID [2020 CRD42020188804], and a PRISMA-Scr Checklist can be found in Supplemental data.

### 2.2 Identifying relevant studies

The team, encompassing experts in PCC, designed a comprehensive search strategy in close collaboration with two expert medical librarians. Literature searches were developed using index terms (e.g., MeSH) and free text words related to PCC, including terms such as person-centred, patient-centred, client-centred, woman-centred, women-centred, child-centred, family-centred, relationship-centred, and people-centred. All variations on term endings, for example, centric, centeredness as well as variations in accompanying terms such as care, practice, approach etc. were included. These terms were chosen based on collective knowledge and experience of the team at that point in time. No time restriction was applied but the language was restricted to English.

The databases PubMed, Scopus, PsychINFO, Cumulative Index to Nursing and Allied Health Literature (CINAHL) and Web of Science were used to retrieve relevant literature. Search terms were adapted according to the different databases. The detailed search syntaxes used in PubMed can be found in Supplemental data. Database searches were conducted on three occasions, with the final search conducted in June 2023. Searches in Grey literature databases or manual searches of reference lists for the included citations were not conducted, and the quality of the literature was not assessed.

### 2.3 Study selection

To be included in the review, the citations needed be published in a scientific journal and (1) include PCC as a concept in the main aim or focus (independent of specific term used) and (2) include an elaborated discussion of the concept used either by: (a) including philosophical, ethical, and theoretical aspects of the concept, or (b) explicitly mentioning the elicitation of a patient narrative and patient-staff partnership.

In this study, the second criterion (2b) was guided by a definition of PCC in which the patient's will, needs and desires are elicited and acknowledged and incorporated into a collaborative partnership involving patient, healthcare professionals and other people of importance in the patient's life. This general, and for our study, guiding definition is in line with the University of Gothenburg Centre for person-centred care (GPCC) framework, first presented by Ekman et al. (4, 5).

All reference types in scientific journals were eligible for inclusion, for example, original quantitative and qualitative studies, reviews, research in brief, editorial letters, study protocols, discussion papers and comments. Citations from all healthcare settings were eligible, for example, neonatal care, paediatric care, child and adolescent healthcare, school healthcare, primary care, hospital care, rehabilitation, residential care, medical home care, home care, hospice care, and education for healthcare professionals and students.

Exclusion criteria were citations not in English, not involving human subjects, citations not using PCC as a concept in the main aim or focus, citations focused solely on shared decision-making, narrative medicine, or person-centred psychotherapy, and citations using a PCC term without explicating and developing what is meant by the term/concept used. We also excluded books, book chapters, theses/dissertations, conference abstracts/proceedings/posters, erratums, and contexts that are not healthcare settings, such as criminal care, social services and general pedagogics/education.

We conducted a stepwise screening and selection process, including both manual screening and a computer software assisted methodology. A random sample of 5,455 citations from the first database searches was selected. The number of citations for the initial set selected was deemed to be a sufficiently large sample, using previous studies as a reference (22). This sample (screening set 1) was imported into Rayyan (23) and the title and abstract of each citation was screened manually by two reviewers independently against inclusion- and exclusion criteria. Citations were classified as “included”, “maybe” or “excluded”. All citations labelled as “maybe” were screened in full text (also by two reviewers), and then classified as included or excluded. This specific step was taken to safeguard that all citations labelled as “included” were relevant.

The classified citations from the manual screening were used to train a predictive classifier model, which was then applied to the remaining citations from the database searches. Having successfully used manually built classifier models in previous work, we tried this option first (22).

Developed by expert language technologists, the model was manually built on single-word frequencies. However, enhancing the precision of this manual model proved time-consuming, prompting us to explore the option of using ready-made screening software.

A bespoke classifier model was built in EPPI-Reviewer 6, which is a software developed and managed by the Evidence for Policy & Practice Information Centre based at University College London (24). Just like our manually built model, this model was built on word frequencies, but instead of single-word frequencies it uses a tri-gram “bag of words' approach, meaning word pairs and triplets are also recognised and counted for each record. In order to validate this methodological change, we conducted a comparison between the models (25). This comparison showed that, using the same set of citations for training, the classifier model built in EPPI-Reviewer could identify relevant citations earlier in the process than the manually built classifier.

All data from screening already conducted while building the manual model (Screening set 1–4) was imported into EPPI-Reviewer's bespoke classifier model, which ranked the remaining citations on their probability of being included in the review. To further strengthen the precision of the model, five additional rounds of screening (set 5–8) were then conducted in EPPI-Reviewer. These rounds also included the ranking of new literature published after the initial searches. After screening all citations ranked as most highly relevant by the classifier model (i.e., all records labelled 89–100), we decided to stop screening. This limitation will be acknowledged in the discussion.

All citations with titles and abstracts that seemed to meet the inclusion criteria were imported and read in full, apart from those which had already been read in full after first being labelled as “maybe” because these had already been marked as “included” or “excluded”. The reviewers resolved any disagreements through discussion and, if needed, consulted with an additional person. Reasons for excluding citations were noted down.

### 2.4 Charting the data

Data relevant to answering the research questions was extracted in accordance with Arksey and O'Malley's (18) framework and entered into NVivo (26). A uniform charting approach was used for all studies included in the review, with data including title, authors, year of publication, country of first author, term used in full text publication, target group, healthcare area and reference type. For empirical studies, the research approach, setting, study design and study population were also extracted. A code-book can be found in Supplemental data. The data-extraction from NVivo was later exported into EPPI-Reviewer and can be found in EPPI-Visualiser (which is a feature in EPPI-reviewer).

A bibliometric analysis was conducted to explore potential research collaborations and clusters in the sample. The analysis was conducted on the citations available in the database Scopus: *n* = 1,150 of the 1,351 included citations. The software VOSviewer (27) and the R package Bibliometrix (28) was used to extract and visualize the co-occurrence of universities/research institutions and keywords in included publications. The keywords used in the publications citing the included publications where also extracted and visualized, this to explore how the field might evolve over time. A more detailed description of this methodology can be found in Supplemental data.

## 3 Results

**Figure 1:**
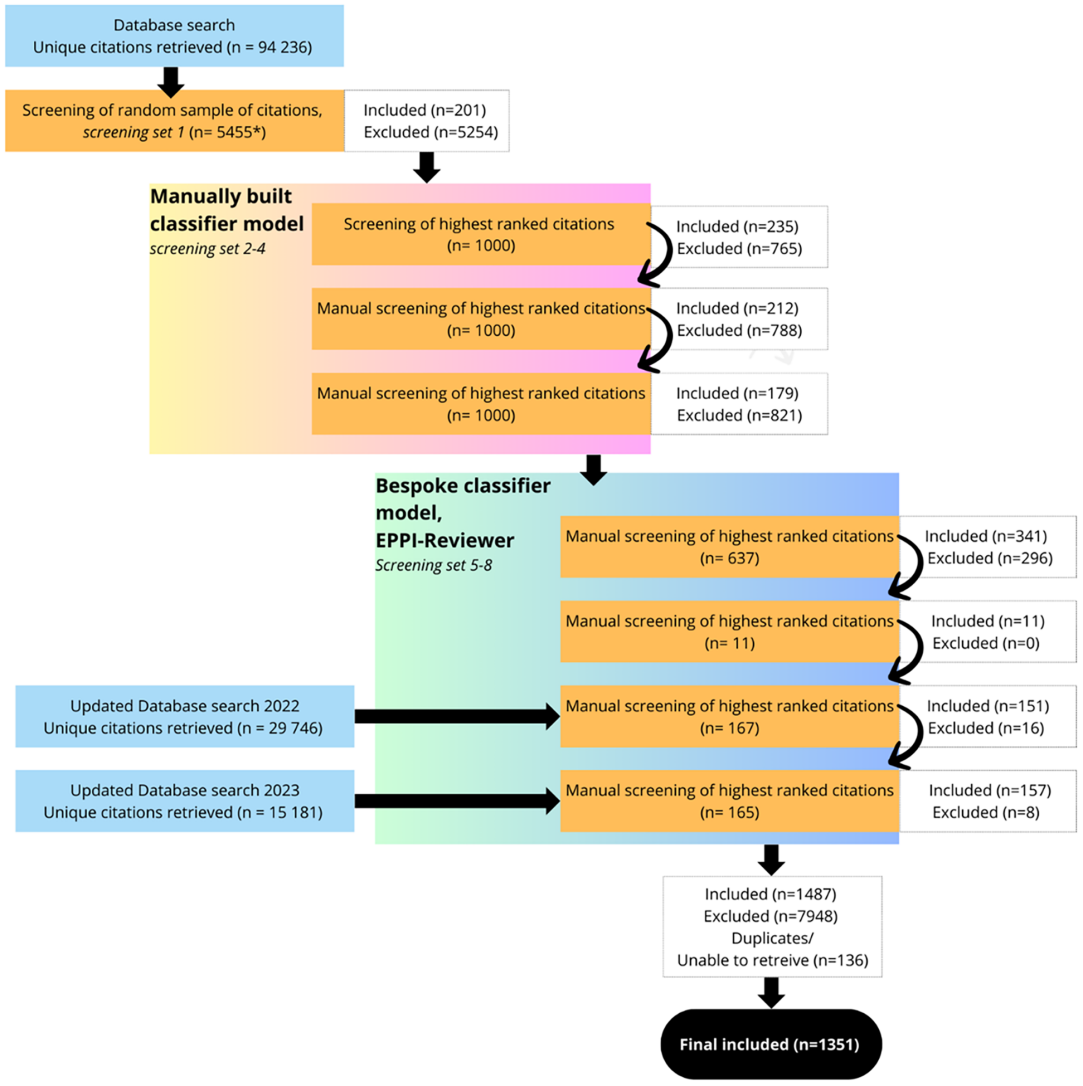
Flowchart of data screening and selection process. *The reason for the uneven number was that after the first 5,000 were selected, 455 were added after a complimentary search on people-centred care, which was not included in the initial search syntax; Full texts were excluded for the following reasons: wrong language, wrong publication type, wrong setting, PCC not the main focus, PCC not explicated or Unable to access.

In total 1,351 citations were included in this scoping review (Figure 1). The results are presented below in narrative summaries, as well as in figures and tables.

### 3.1 Populations, settings, research approaches and designs

Most publications were focused on adults or did not specify any target group (*n* = 925). Children and the elderly were evenly distributed thereafter, representing *n* = 217 and *n* = 218 publications respectively (Table 1). General in-patient and out-patient care is the largest category of healthcare area within our data (*n* = 836). The next largest healthcare category is publications with an unspecified healthcare area i.e., literature for which no healthcare area or context is explicitly mentioned, for example, an explicitly theoretical article (*n* = 257).

**Table 1:** Characteristics of included publications 1972–2023 (June).

Included publications (*n* = 1,351)	Total *n* (%)	Empirical studies (*n* = 658)	Total *n* (%)
Target group^a^		Research approach of empirical studies	
Adults/unspecified	925	Qualitative	303 (46.0)
Children	218	Quantitative	281 (42.7)
Elderly	217	Mixed-methods	74 (11.2)
Healthcare area^a^		Setting of empirical studies^a^	
General in-patient and out-patient care^b^	836	Hospital care (specialist care)	329
Elderly, long term, residential, hospice	130	Residential home care	102
Psychiatric care	48	Primary care	96
Health promotion	36	Healthcare student education	42
Rehabilitation, habilitation, disability	16	Home care	31
Home care	13	Unspecified^e^	44
Dentistry	12	Other^f^	59
Unspecified^c^	257		
Other^d^	18	Study design of empirical studies^a^	
		Descriptive, exploratory, interpretive	427
Reference type		Quality improvement study	44
Editorials, letters, commentaries, anecdotes	90 (6.7)	Quasi experimental	34
Empirical studies	658 (48.7)	Participatory, action research	32
Literature reviews	163 (12.1)	Experimental (randomisation)	25
Study protocols	16 (11.8)	Case study	18
Theoretical studies	424 (31.4)	Other^g^	80
		Study population of empirical studies^a^	
		Patients	274
		Health professionals	370
		Family, parents, significant others	128
		Students	40
		Other^h^	76

^a^ More than one category can be coded in citations.

^b^ Includes a variety of in-patient and out-patient healthcare areas.

^c^ Citations not stating specific area.

^d^ Includes for example chiropractic care and pharmaceutical care.

^e^ No specific setting stated.

^f^ Includes for example. Rehabilitation and audiology.

^g^ Includes for example development and validation of questionnaires.

^h^ Includes for example hospital managers and members of the public. See additional details in Supplemental data.

**Figure 2:**
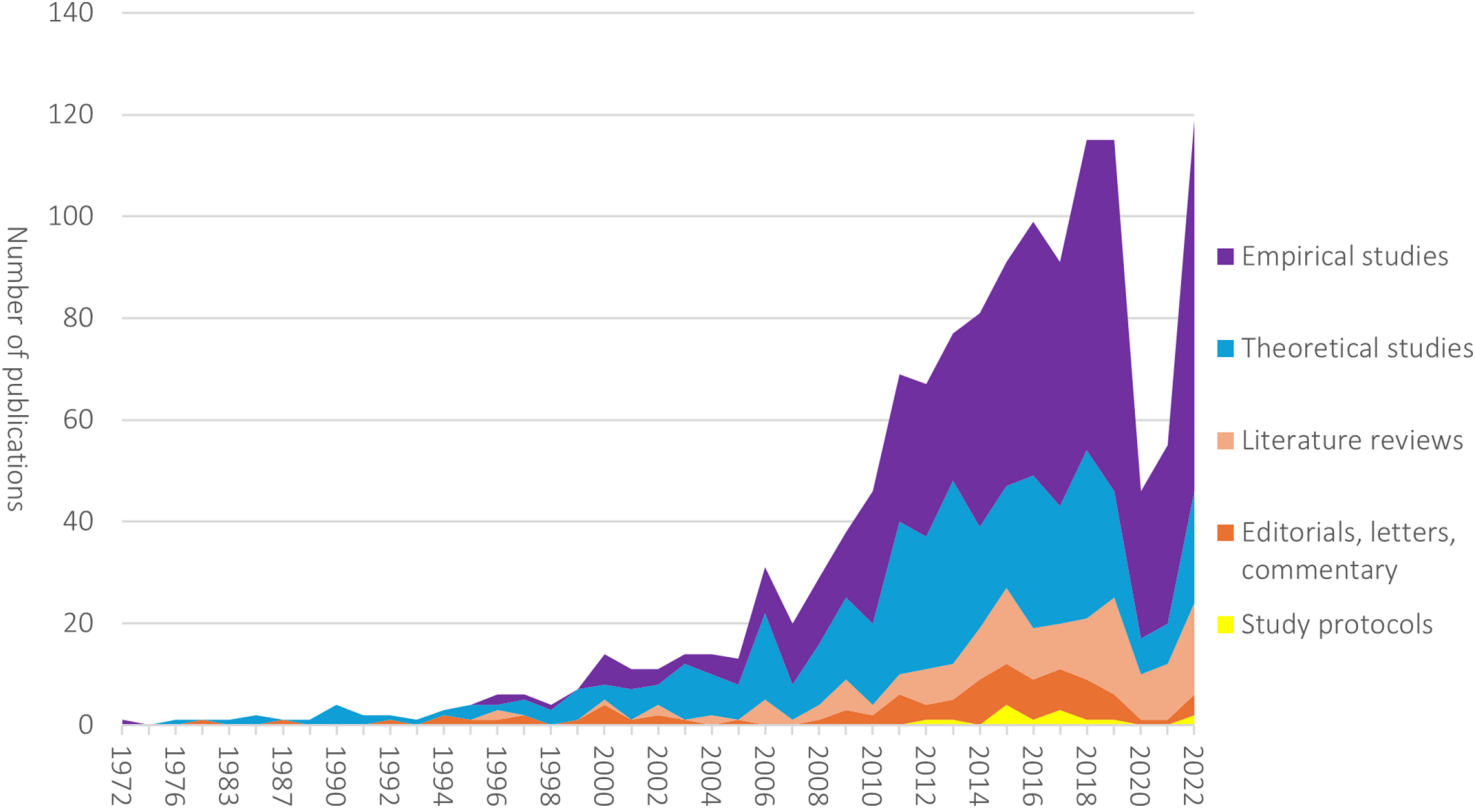
Development over time in number of publications in each reference type, 1972–2022.

Empirical studies made up the majority of the sample (*n* = 658), followed by theoretical studies (*n* = 424), literature reviews (*n* = 163) and editorials/letters/commentaries and anecdotal publications (*n* = 90). Study protocols were the smallest group (*n* = 16). Looking at development over time, theoretical and empirical studies have followed the same path, sharing the top spot for reference type until 2013 (Figure 2), followed thereafter by an upswing in empirical studies. In recent years, empirical studies have become the predominant publication type, but the number of literature reviews has also increased.

The research approach of the empirical studies in our sample was most often either qualitative (*n* = 303, 46%) or quantitative (*n* = 281, 43%). The predominant setting was hospital care (*n* = 329) and has remained so over the years, followed by residential home care (*n* = 103). The study design was most often descriptive/exploratory/interpretive (*n* = 427), and the most common study population was health professionals (*n* = 370) or patients (*n* = 274), which is also the case looking at development over time. No clear increase in research focused on other groups can be seen in our data.

### 3.2 PCC terms and keywords

The most frequently used term within our data is patient-centred (*n* = 539, 40%), followed by person-centred (*n* = 425, 31%), family-centred (*n* = 240, 18%), and patient and family-centred care (*n* = 68, 5%) (Table 2). Other terms used are client-centred, woman-centred, people-centred and relationship centred. Multiple terms within one publication were also used.

**Table 2:** Term used.

Term used	Total *n* (%)
Patient-centred^a^	539 (39.9)
Person-centred	425 (31.5)
Family-centred	240 (17.8)
Patient and family-centred	68 (5.0)
Client-centred	15 (1.1)
Woman-centred	10 (0.7)
People-centred	10 (0.7)
Relationship-centred	10 (0.7)
Multiple terms^b^	21 (1.6)
Other^c^	13 (1.0)

^a^ Includes citations using the term *patient centric*.

^b^ Includes citations using multiple terms, such as patient-centred and person-centred.

^c^ Includes child and family-centred, child-centred, community-centred, person and family-centred, person- and relationship-centred, resident-centred, student-centred, and soldier-centred.

**Figure 3:**
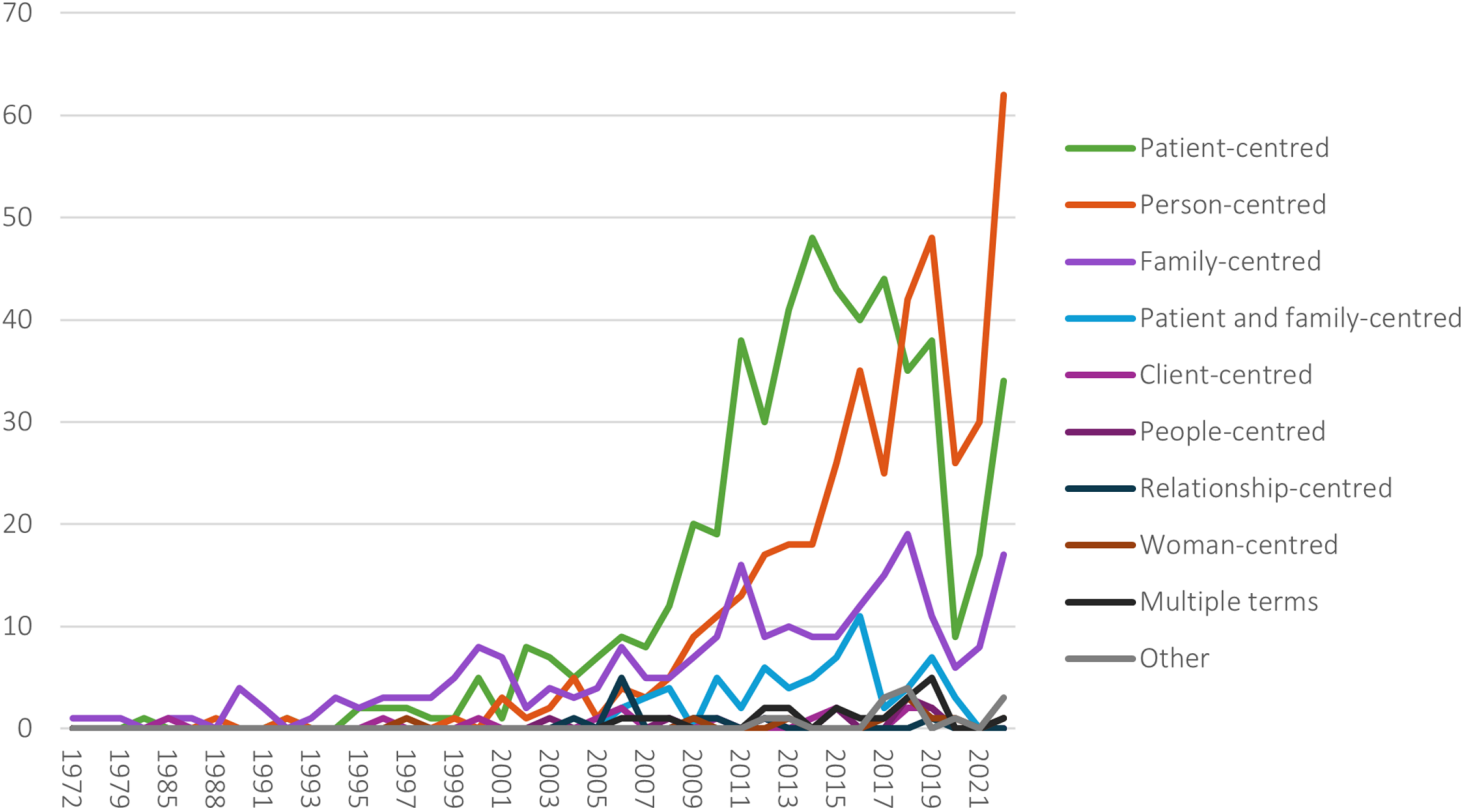
Development in use of terms over time, 1972–2022.

Exploring development over time, patient-centred care was the dominant term used in our sample until 2018, when the term person-centred care took the lead (Figure 3). In our sample, the term family-centred care saw an increase in use during the 1990s, and has had a small, but steady growth over time. Other combined centredness terms have emerged, such as patient- and family-centred care.

**Figure 4:**
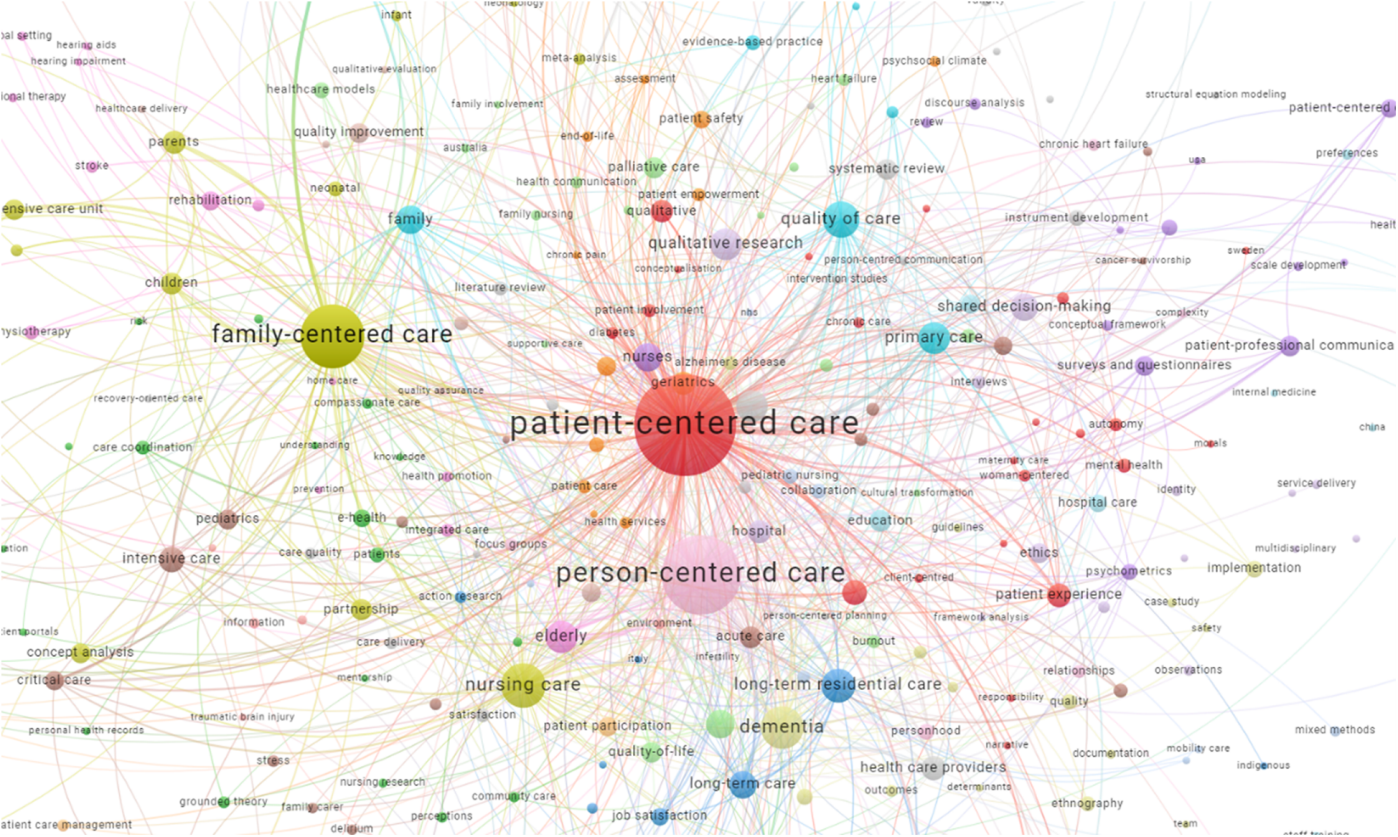
Keywords used in included publications. https://tinyurl.com/2n4daszo. The map is based on keywords with a minimum occurrence of 2 in the included publications.

We performed bibliometric analysis to explore keywords used within the included publications. Apart from PCC terms, the ten keywords most often used were nursing care, dementia, quality of care, long-term residential care, elderly, communication, primary care, qualitative research, family and nurses (Figure 4).

**Figure 5:**
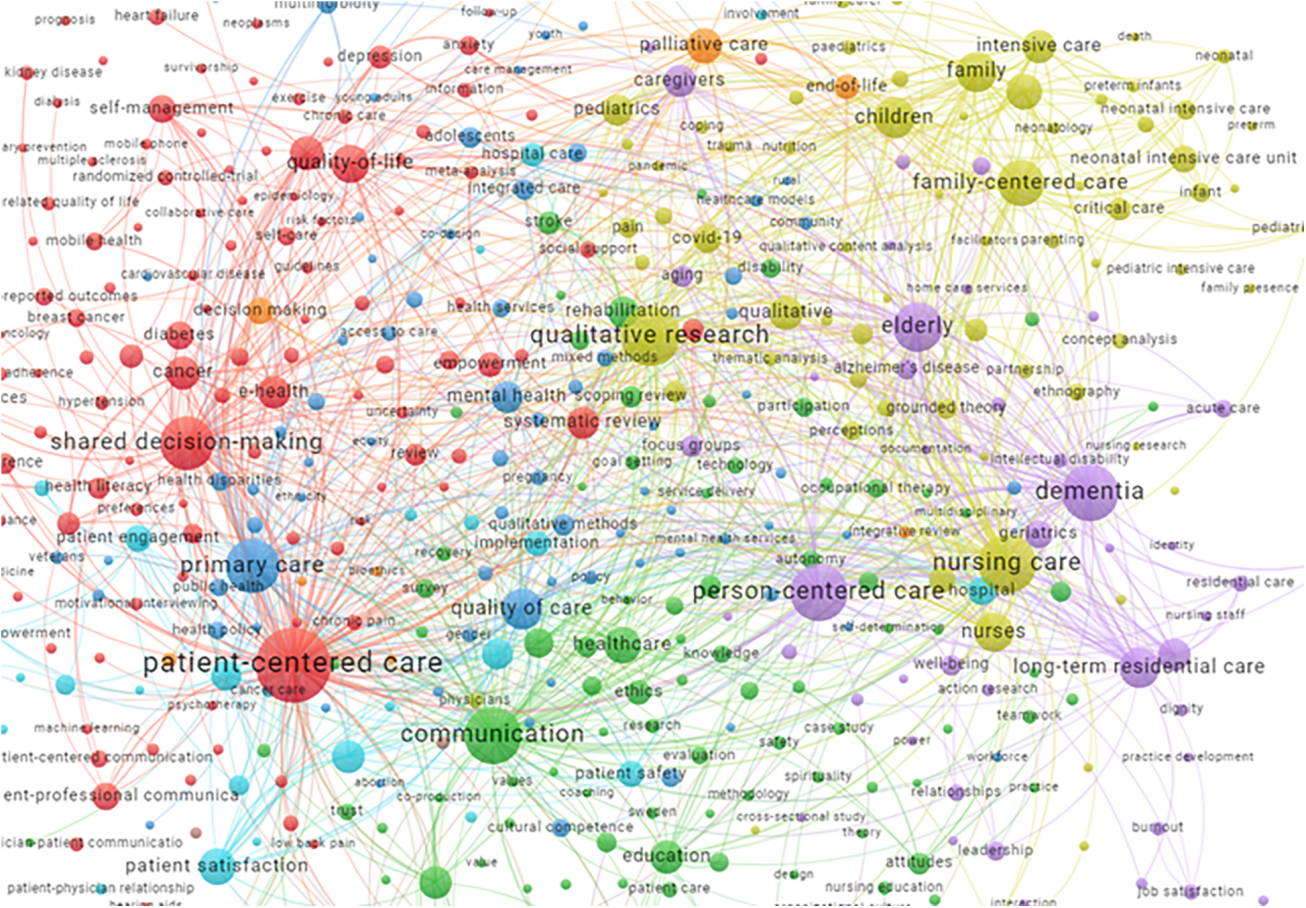
Keywords used in publications citing included publications (top 500). https://tinyurl.com/2z6t2bwm.

While exploring publications that cited the included publications (Figure 5), we saw that the most frequently used keywords (apart from PCC terms) were more or less the same, namely, qualitative research, nursing care, communication, dementia, primary care, shared decision-making, elderly, children, quality of care and family.

### 3.3 Research collaborations and clusters

**Figure 6:**
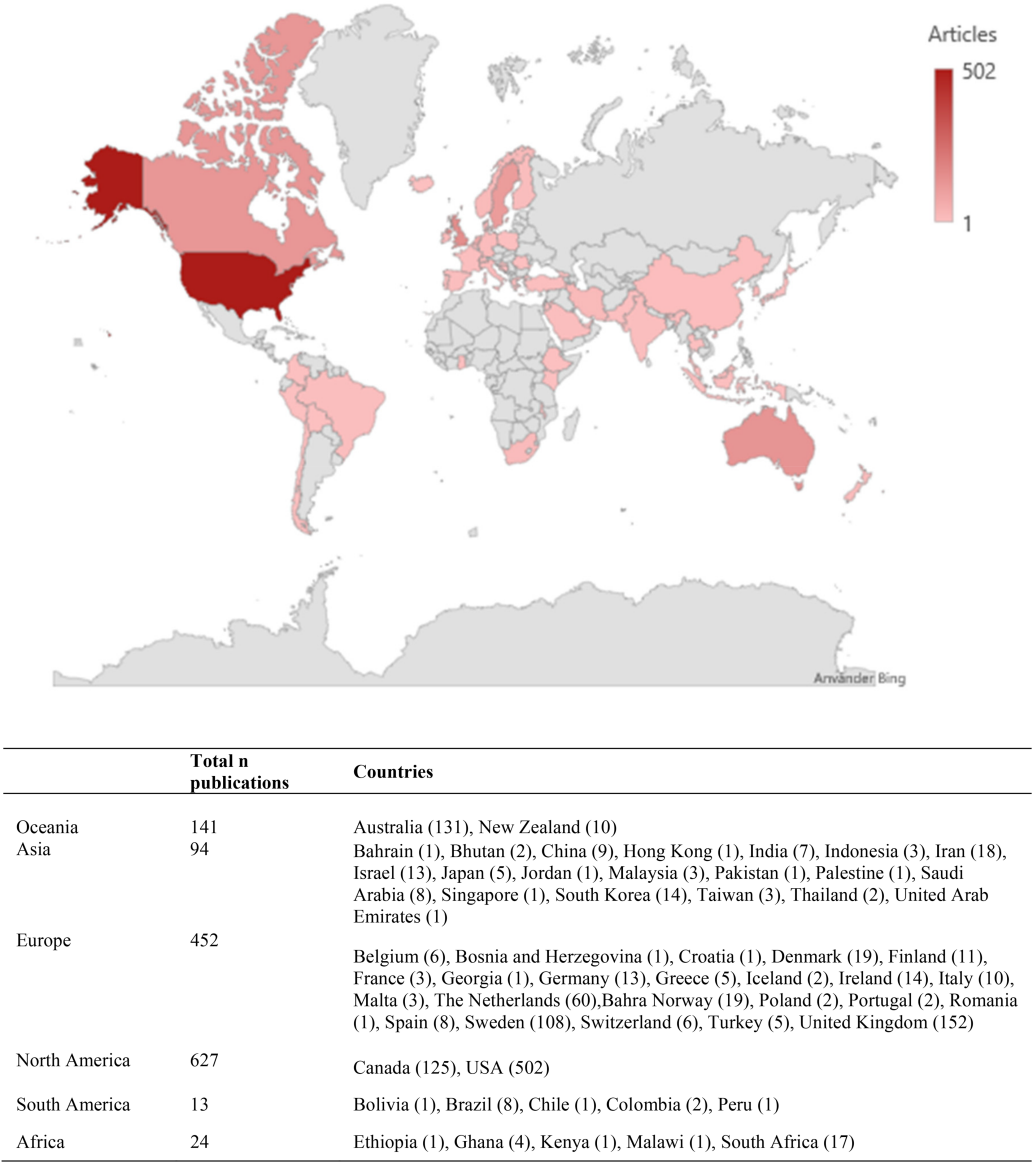
Geographical distribution.

Six continents were represented in our sample of research on PCC, with most publications from the United States (*n* = 502), United Kingdom (*n* = 152), Australia (*n* = 131), Canada (*n* = 125) and Sweden (*n* = 108), see Figure 6.

#### 3.3.1 Universities/research institutions

**Figure 7:**
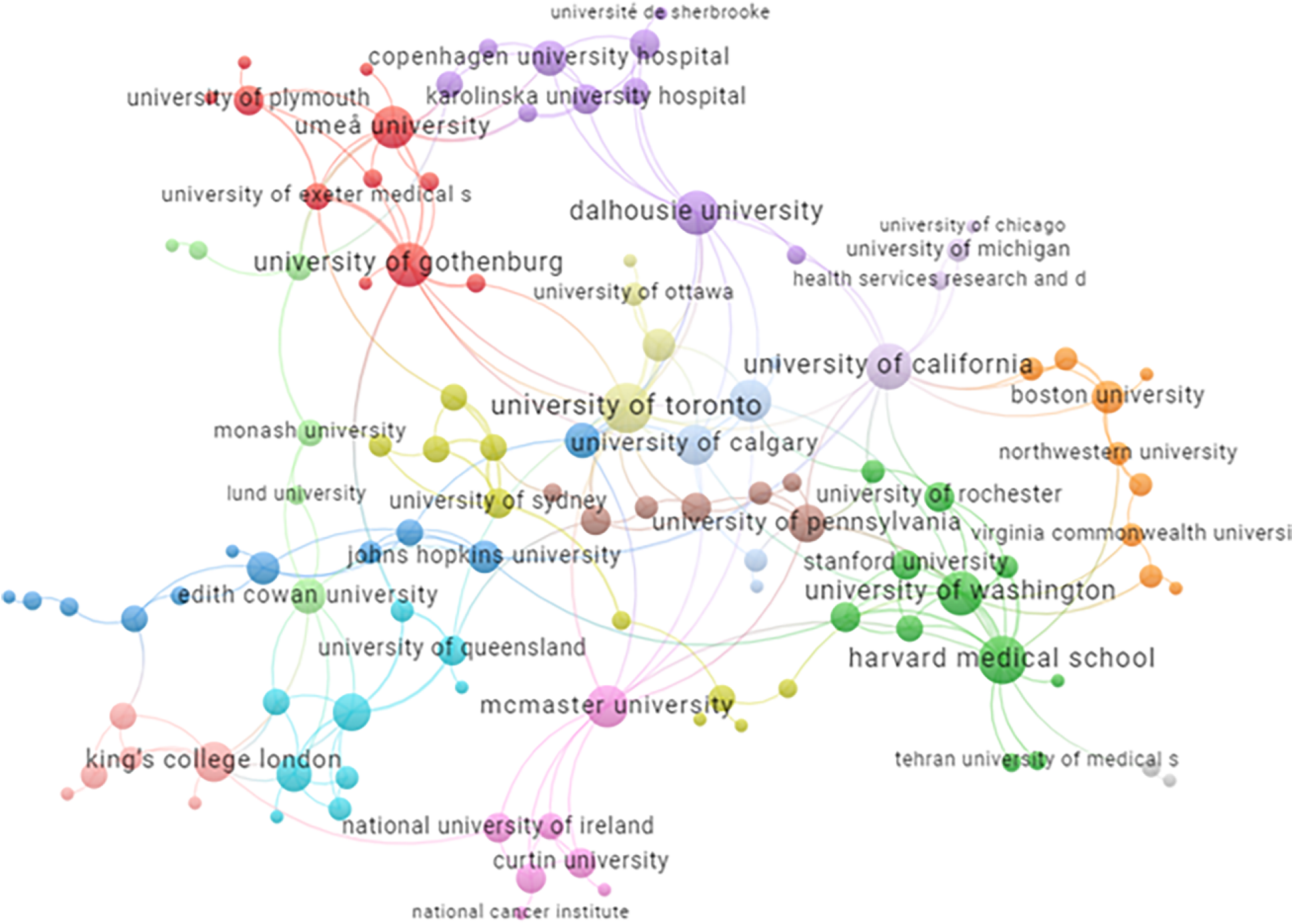
Universities/research institutions in included publications. https://tinyurl.com/2q9knonr. This map is based on institutions with at least five shared publications with another institution.

Several clusters of universities/research institutions appear in the analysis (see colours in Figure 7).

## 4 Discussion

This scoping review provides an overview of the large and not easily delimited field of research on PCC. The terms patient-centred care, person-centred care and family-centred care were the most used within our whole sample. Person-centred care was the most used term after 2017. Combined terms, such as patient- and family-centred care have also come to the fore in recent years, and could potentially be traced to more groups taking on the PCC terminology. Some terms, e.g., woman-centred care, were exclusively used within a specific field—in this case, midwifery—which explains the limited number of publications represented in the sample.

PCC research is being conducted globally, with representation from researchers across six continents. It is nevertheless important to keep in mind that PCC is discussed as an approach that evolved in high-income countries, and therefore data across low-income and middle-income countries are limited (29). The top six countries represented in this review are the US, UK, Australia, Canada, Sweden, and the Netherlands. Comparing the top countries in this review to overall research output (30), the US, UK, Australia, and Canada all rank highly, while Sweden and the Netherlands are further down the list, potentially suggesting a specific interest or incentive for PCC research in the latter two countries.

Our sample clearly shows an increasing focus on empirical studies, as well as reviews, indicating that the field is in some ways maturing. There are various ways of defining a mature research field, but Keathley-Herring et al. (31) propose that an important aspect is that the field is put into practice, which the increase in empirical studies may suggest.

Nevertheless, the extent to which the number of empirical publications translates to actual implementation of PCC practices is unclear. Rosengren et al. (32, 33) suggest that PCC implementation in Europe depends on the healthcare system model. Countries with tax-funded public healthcare systems (Beveridge model), such as the UK and Scandinavian countries, may have been more successful in diffusing PCC than those with statutory health insurance-based systems (Bismarck model) like Germany, Austria, Switzerland and the Czech Republic.

Another factor pointing towards a mature research field, as discussed by Keathley-Herring and colleagues (31), is that the field is broadly accessible and agreed upon by a distinct research community. The bibliometric analysis in our study revealed collaborations between research groups and universities, which could suggest an emerging research community. However, there are also factors indicating that PCC is still only a moderately mature field, as there is no robust differentiation from other research areas. As previously discussed, there is a lack of consensus on the PCC concept, which suggests that the field is continuing to evolve (34). Even if this could be seen as positive, the lack of clarity in conceptualization and terminology presents barriers to a comprehensive and detailed overview of the field.

### 4.1 Method discussion and limitations

This project has been a challenge in many ways, and for us involved a methodological journey. Choosing to include a large variety of terms in our search syntax resulted in a large quantity of publications, meaning we had to make a number of decisions which could impact the overall results.

Firstly, due to the large number of publications, we did not include manual searches, which can be seen as a limitation. Secondly, we chose only to include citations explicitly focused on PCC in main aim and focus, as we did not want records which solely used a term without explicit discussion on the construct behind it. Thus, publications that were relevant but only used the term, without explaining its grounding according to our criteria, were excluded. Thirdly, we chose to use text mining features to assist the screening process and decided to end screening after we had gone through all records deemed most relevant by the classifier model (placed in the pile of a likelihood of 89–100 in EPPI-Reviewer). This could result in many potentially relevant citations being excluded from the complete sample.

Another factor which can be seen as a limitation is that our definition of PCC was general but nevertheless partly guided by the Gothenburg framework (4, 35), which can be seen as inherent bias in screening. There is a possibility that some citations related to, for example, people-centred care and family-centred care were excluded in our database queries and screening protocol. We chose to focus on the personal narrative and partnership as one part of our inclusion criteria, which could have excluded records more focused on the community or family perspectives, for example. The term people-centred care focuses on the macro perspective of communities, which was not spotlighted in our criteria for inclusion. Possible additional specific terms, such as LGBTQ-centred, were not included in our search syntax, which in hindsight could also be seen as a limitation.

As for the bibliometric analysis based on Scopus data, covering 1,150 of the 1,351 publications included in the review, there is potential risk that relevant keywords and universities/research institutions occurring in the total data set might be missing in the figures presented.

Our approach made the project very time consuming, as well as labour intensive. This has implications, as the large number of people involved at various stages of the project could introduce rater bias in the screening process. The process of categorising areas of research literature also comes with limitations. We aimed to create categories of characteristics that would include most of the studies, but for some studies we had to create an “unspecified” category, as well as an “other” category. Another limitation is that the countries represented in our study have different systems of healthcare and care organization, meaning there is not necessarily a perfect fit with our categorization of healthcare area and settings.

### 4.2 Conclusion and implications

This review presents an overview of the literature showing that PCC research is being conducted worldwide in international collaborations. Most included publications use the terms *patient*, *person*, or *family centred care*. The term *person-centred care* is most frequently used in recent publications. Most publications are empirical studies of adult patients or professionals within a hospital care setting.

Our study demonstrates that using a broad conceptualization of PCC research results in the inclusion of a wide variety of terms. Such a variety of terms results in a large amount of citations, which subsequently affects how far one can present a comprehensive and detailed overview of the literature. While our study does not provide an answer as to how to manage these barriers, it does point to the necessity of making methodological choices clear, which will help prevent fragmentation of knowledge in future studies attempting towards PCC research synthesis.

This result, apart from working as a call for action for researchers in PCC to be more transparent in choice of methodology, could also be of interest for research in other fields encompassing substantial amounts of literature with more than one term applied, overlapping concepts, and that is not easily delimited.

## Data Availability

The included publications and their coding can be retrieved in EPPI-Reviewer Visualizer https://eppi.ioe.ac.uk/eppi-vis/login/open?webdbid=309.

## References

[1] Sheikh K Ranson MK Gilson L. Explorations on people centredness in health systems. Health Policy Plan. (2014) 29:1–5. 10.1093/heapol/czu082 25274634 PMC4202918

[2] McCormack B. The person-centred nursing and person-centred practice frameworks: from conceptual development to programmatic impact. Nurs Stand. (2020) 35(10):86–9. 10.7748/ns.35.10.86.s40

[3] McCormack B McCance T Martin S McMillan A Bulley C. Fundamentals of Person-centred Healthcare Practice. Oxford: Wiley (2021).

[4] Ekman I Swedberg K Taft C Lindseth A Norberg A Brink E Person-centered care–ready for prime time. Eur J Cardiovasc Nurs. (2011) 10(4):248–51. 10.1016/j.ejcnurse.2011.06.008 21764386

[5] Ekman I. Practising the ethics of person-centred care balancing ethical conviction and moral obligations. Nurs Philos. (2022) 23(3):e12382. 10.1111/nup.12382 35213781 PMC9285079

[6] Santana MJ Manalili K Jolley RJ Zelinsky S Quan H Lu M. How to practice person-centred care: a conceptual framework. Health Expect. (2018) 21(2):429–40. 10.1111/hex.12640 29151269 PMC5867327

[7] European Committee for Standardization. Patient involvement in health care—minimum requirements for person-centred care. EN 17398:2020. CEN-CENELEC Management Centre; (2020).

[8] World Health O. WHO Global Strategy on People-Centred and Integrated Health Services: Interim Report. Geneva: World Health Organization (2015).

[9] Nkhoma KB Cook A Giusti A Farrant L Petrus R Petersen I A systematic review of impact of person-centred interventions for serious physical illness in terms of outcomes and costs. BMJ Open. (2022) 12(7):e054386. 10.1136/bmjopen-2021-054386 35831052 PMC9280891

[10] Giusti A Nkhoma K Petrus R Petersen I Gwyther L Farrant L The empirical evidence underpinning the concept and practice of person-centred care for serious illness: a systematic review. BMJ Glob Health. (2020) 5(12):e003330. 10.1136/bmjgh-2020-003330 33303515 PMC7733074

[11] Hughes JC Bamford C May C. Types of centredness in health care: themes and concepts. Med Health Care Philos. (2008) 11(4):455–63. 10.1007/s11019-008-9131-5 18398697

[12] Håkansson Eklund J Holmström IK Kumlin T Kaminsky E Skoglund K Höglander J Same same or different?” A review of reviews of person-centered and patient-centered care. Patient Educ Couns. (2019) 102(1):3–11. 10.1016/j.pec.2018.08.029 30201221

[13] National Center for Biotechnology Information (NCBI). Bethesda (MD): National Library of Medicine (US), National Center for Biotechnology Information; (1988). Available online at: https://www.ncbi.nlm.nih.gov/mesh/?term=patient-centered+care (cited July 2, 2024).

[14] Nolte E Anell A. Person-centred health systems: strategies, drivers and impacts. In: Nolte E Merkur S Anell A, editors. Achieving Person-Centred Health Systems: Evidence, Strategies and Challenges. European Observatory on Health Systems and Policies. Cambridge: Cambridge University Press (2020). p. 41–74.

[15] Berntsen G Chetty M Ako-Egbe L Yaron S Phan Thanh P Castro I Report No.: ISBN 978-0-9955479-2-6. Person-Centred Care Systems: From Theory to Practice. A White Paper for ISQUA. (2022).

[16] Sturgiss EA Peart A Richard L Ball L Hunik L Chai TL Who is at the centre of what? A scoping review of the conceptualisation of ‘centredness’ in healthcare. BMJ Open. (2022) 12(5):e059400. 10.1136/bmjopen-2021-059400 35501096 PMC9062794

[17] Grant MJ Booth A. A typology of reviews: an analysis of 14 review types and associated methodologies. Health Info Libr J. (2009) 26(2):91–108. 10.1111/j.1471-1842.2009.00848.x 19490148

[18] Arksey H O'Malley L. Scoping studies: towards a methodological framework. Int J Soc Res Methodol. (2005) 8(1):19–32. 10.1080/1364557032000119616

[19] Levac D Colquhoun H O'Brien KK. Scoping studies: advancing the methodology. Implement Sci. (2010) 5:69. 10.1186/1748-5908-5-69 20854677 PMC2954944

[20] Moher D Shamseer L Clarke M Ghersi D Liberati A Petticrew M Preferred reporting items for systematic review and meta-analysis protocols (PRISMA-P) 2015 statement. Syst Rev. (2015) 4(1):1. 10.1186/2046-4053-4-1 25554246 PMC4320440

[21] Tricco AC Lillie E Zarin W O'Brien KK Colquhoun H Levac D PRISMA Extension for scoping reviews (PRISMA-ScR): checklist and explanation. Ann Intern Med. (2018) 169(7):467–73. 10.7326/M18-0850 30178033

[22] Sawatzky R Porterfield P Lee J Dixon D Lounsbury K Pesut B Conceptual foundations of a palliative approach: a knowledge synthesis. BMC Palliat Care. (2016) 15:5. 10.1186/s12904-016-0076-9 26772180 PMC4715271

[23] Ouzzani M Hammady H Fedorowicz Z Elmagarmid A. Rayyan-a web and mobile app for systematic reviews. Syst Rev. (2016) 5(1):210. 10.1186/s13643-016-0384-4 27919275 PMC5139140

[24] Thomas J Graziosi S Brunton J Ghouze Z O'Driscoll P Bond M EPPI-Reviewer: Advanced Software for Systematic Reviews, Maps and Evidence Synthesis. University College London: EPPI Centre, UCL Social Research Institute (2023).

[25] Forsgren E Wallström S Feldthusen C Zechner N Sawatzky R Öhlén J. The use of text-mining software to facilitate screening of literature on centredness in health care. Syst Rev. (2023) 12(1):73. 10.1186/s13643-023-02242-0 37120578 PMC10148558

[26] Dhakal K. NVivo. J Med Libr Assoc. (2022) 110(2):270–2. 10.5195/jmla.2022.1271 35440911 PMC9014916

[27] van Eck NJ Waltman L. Software survey: VOSviewer, a computer program for bibliometric mapping. Scientometrics. (2010) 84(2):523–38. 10.1007/s11192-009-0146-3 20585380 PMC2883932

[28] Aria M Cuccurullo C. Bibliometrix: an R-tool for comprehensive science mapping analysis. J Informet. (2017) 11(4):959–75. 10.1016/j.joi.2017.08.007

[29] Giusti A Pukrittayakamee P Alarja G Farrant L Hunter J Mzimkulu O Developing a global practice-based framework of person-centred care from primary data: a cross-national qualitative study with patients, caregivers and healthcare professionals. BMJ Glob Health. (2022) 7(7):e008843. 10.1136/bmjgh-2022-008843 35831035 PMC9280875

[30] Schneider BAJ Thomas P. Publications Output: U.S. Trends and International Comnparisons. NCSES. National Center for Science and Engineering Statistics, Directorate for Social, Behavioral and Economic Sciences; (2023).

[31] Keathley-Herring H Van Aken E Gonzalez-Aleu F Deschamps F Letens G Orlandini PC Assessing the maturity of a research area: bibliometric review and proposed framework. Scientometrics. (2016) 109:927–51. 10.1007/s11192-016-2096-x

[32] Rosengren K Brannefors P Carlstrom E. Adoption of the concept of person-centred care into discourse in Europe: a systematic literature review. J Health Organ Manag. (2021) 35(9):265–80. 10.1108/JHOM-01-2021-0008 34523306 PMC9136870

[33] Rosengren K Buttigieg SC Badanta B Carlstrom E. Diffusion of person-centred care within 27 European countries—interviews with managers, officials, and researchers at the micro, meso, and macro levels. J Health Organ Manag. (2022). 10.1108/JHOM-02-2022-0036 36367331

[34] Mitchell P Cribb A Entwistle V. Vagueness and variety in person-centred care. Wellcome Open Res. (2022) 7:170. 10.12688/wellcomeopenres.17970.1 35865218 PMC9277200

[35] Britten N Ekman I Naldemirci Ö Javinger M Hedman H Wolf A. Learning from Gothenburg model of person centred healthcare. Br Med J. (2020) 370:m2738. 10.1136/bmj.m2738 32873594

[36] Pollock A Campbell P Struthers C Synnot A Nunn J Hill S Development of the ACTIVE framework to describe stakeholder involvement in systematic reviews. J Health Serv Res Policy. (2019) 24(4):245–55. 10.1177/1355819619841647 30997859

