# Supplementary material for: Person-centred care as an evolving field of research: a scoping review
Source: Front Health Serv. 2025 Apr 4;5:1534178. doi: 10.3389/frhs.2025.1534178 (PMC12006168; doi:10.3389/frhs.2025.1534178)
Supplement: Supplementary file 1 [file Datasheet1.pdf]

Supplementary Data

1. Search terms for Pubmed

((“personcent*”[tiab]) OR (“person-cent*”[tiab]) OR (“person cent*”[tiab]) OR (“patientcent*”[tiab]) OR (“patient-cent*”[tiab]) OR (“patient cent*”[tiab]) OR (“Patient-Centered Care”[Mesh]) OR (“clientcent*”[tiab]) OR (“client-cent*”[tiab]) OR (“client cent*”[tiab]) OR (“relationshipcent*”[tiab]) OR (“relationship-cent*”[tiab]) OR (“relationship cent*”[tiab]) OR (“womencent*”[tiab]) OR (“women-cent*”[tiab]) OR (“women cent*”[tiab]) OR (“womancent*”[tiab]) OR (“woman-cent*”[tiab]) OR (“woman cent*”[tiab]) OR (“familycent*”[tiab]) OR (“family-cent*”[tiab]) OR (“family cent*”[tiab]) OR (“childcent*”[tiab]) OR (“child-cent*”[tiab]) OR (“child cent*”[tiab]) OR (“peoplecent*”[tiab]) OR (“people-cent*”[tiab]) OR (“people cent*”[tiab]))

2. Code-book data extraction

1	**Title**		
2	**Author(s)**		
3	**Journal**		
4	**Year**		*Publication date, printed version*
5	**Country**		*First affiliation of first author*
6	**Term used**		*The main term described. Multiple terms possible*
7	**Target group**		*Multiple groups possible*
		Children or adolescents (0-18)	
		Adult/unspecified	*Studies not stating target group are coded as adults/unspecified*
		Elderly (+65)	*includes dementia, nursing homes*
8	**Health care area**		
		Dentistry	
		Long term care	*Includes residential care, hospice*
		Psychiatric care	
		Somatic care	*Includes care in general, primary care, neonatal care, delivery care*
		Health promotion	*No disease. Includes family care planning, maternity care, school health care, business health care*
		Home care	
		Rehabilitation	*Includes habilitation, disability*
		Unspecified	*No specific area, e.g. theoretical with no specific context, health care student education*
		Other	*Includes infertility care, chiropractic care, pharmaceutical care, complementary medicine*
9	**Reference type**		
		Editorials, letters, commentary	
		Theoretical studies	*Includes discussion papers, essay, position papers, conceptual analysis*
		Literature reviews	
		Study protocols	
		Empirical studies*	
*	**Empirical studies**		
	**Research approach**		
		Qualitative	
		Quantitative	
		Mixed methods	
	**Study design**		
		Descriptive, explorative	*Includes Delphi*
		Case study	
		Experimental	*With randomisation*
		Quasi experimental	*No randomisation*
		Participatory, action research	
		Quality improvement	
		Unspecified	
		Other	*Includes e.g. development/adaptation/translation and validation of questionnaires/tools*

**Setting**		
	Home care	
	Hospital care	*Includes all forms of specialist care*
	Health care student education	
	Primary care	*Includes patient centred medical homes*
	Residential home/nursing home	
	Unspecified	*No specific setting, health care in general e.g. national surveys, infra structure issues*
	Other	*Includes services for intellectual disability, rehabilitation, audiology*
**Study population**		*Multiple populations possible*
	Family, parents, significant others	
	Health professionals	
	Patients	
	Students	
	Other	*Includes administrators, managers, politicians, policy makers, citizens*

3. Methodology for the Bibliometric Analysis

Data Selection Strategy

The total set of 1351 publications were exported from EndNote as a BibTex file. A Python script (https://github.com/stinajoss/Bibliometri_GU/blob/main/scoping_review_bibtex.ipynb) was then executed to extract bibliographic identifiers. This data was finally, with some manually work, matched with data from three databases: Scopus, Web of Science, and PubMed.

Out of the 1351 publications:

- 1150 matched with data from Scopus
- 1086 matched with data from PubMed
- 966 matched with data from Web of Science

Scopus was chosen for the bibliometric analysis in this study due to its superior coverage compared to PubMed and Web of Science. The bibliographic data from Scopus was extracted and downloaded as a CSV file in March 2024.

Tools of Analysis

The bibliometric analysis in this study was conducted using VOSviewer (version 1.6.20) and the R package Bibliometrix (version 4.2.3). VOSviewer was utilized to calculate and visualize the co-occurrence of keywords. Bibliometrix was employed to calculate the co-occurrence of author affiliations, and the visualization of this was then created with VOSviewer.

Type of Analysis

The bibliometric analysis conducted in this study included the following:

- Co-occurrence of keywords in the 1150 publications with a threshold of 2 keywords.
- Co-occurrence of keywords in the publications citing the 1150 publications, showing the top 500 most used keywords.
- Co-authorships based on affiliations, with a threshold set to a minimum of 5 publications from an affiliation.

For the keywords, author-keywords were used, which are the keywords used by the authors in the original papers. Since these keywords are not indexed by a controlled vocabulary, synonyms will occur. To handle this, a thesaurus with a list of words to merge was used.

Interactive Maps

Co-occurrence of keywords: https://tinyurl.com/2n4daszo
Co-occurrence of keywords in citing documents: https://tinyurl.com/2z6t2bwm
Co-authorship: https://tinyurl.com/2q9knonr
